# Gastric outlet obstruction in an 11‐year‐old girl: A case report

**DOI:** 10.1002/jpr3.12062

**Published:** 2024-04-01

**Authors:** Ana Sofia Figueiredo, Carolina Soares‐Aquino, Rita Amorim, Lina Melão, Céu Espinheira, Isabel Pinto Pais, Miguel Campos, Eunice Trindade

**Affiliations:** ^1^ Department of Pediatrics Centro Hospitalar Trás os Montes e Alto Douro Vila Real Portugal; ^2^ Department of Pediatric Surgery Centro Hospitalar Universitário São João Porto Portugal; ^3^ Department of Pediatrics Centro Hospitalar Universitário São João Porto Portugal; ^4^ Department of Radiology Centro Hospitalar Universitário São João Porto Portugal; ^5^ Department of Pediatric Gastroenterology Centro Hospitalar Universitário São João Porto Portugal

**Keywords:** endoscopy, pyloric stenosis, pyloroplasty

## Abstract

Pyloric stenosis commonly affects infants and rarely causes gastric outlet obstruction in adolescents and older children. We present the case of an 11‐year‐old girl with a 2‐month history of recurrent postprandial vomiting and weight loss. On physical examination, the patient presented with abdominal distension. Upper gastrointestinal endoscopy revealed a very small pyloric orifice through which the endoscope could not be advanced. Abdominal ultrasonography and a computed tomography confirmed pylorus thickening. She underwent Heineke‐Mikulicz pyloroplasty with symptom resolution.

## 1 INTRODUCTION

Pyloric stenosis is caused by thickening of the pylorus muscle, leading to gastric outlet obstruction (GOO). This condition is commonly seen in infants during the first 1–3 months of life (incidence of 1.5–3 per 1000 live births) and is known as infantile hypertrophic pyloric stenosis. However, pyloric stenosis presenting beyond infancy is markedly uncommon, and few cases have been described in adults, older children or adolescents.^1^, ^2^

Here, we present the case of an 11‐year‐old girl who was successfully treated for late presentation of hypertrophic pyloric stenosis.

## 2 CASE REPORT

An 11‐year‐old girl was admitted at the pediatric emergency department with multiple episodes of non‐bilious and non‐bloody post prandial vomiting and weight loss for 2 months. There was no history of fever or diarrhea. Upon physical examination, she presented with pallor, thin, and with abdominal distension.

Prior the admission, the patient had regular pneumology and endocrinology appointments due to recurrent pulmonary infections and short stature. She was treated with montelukast, inhaled salmeterol/fluticasone, and subcutaneous growth hormone.

**Figure 1:**
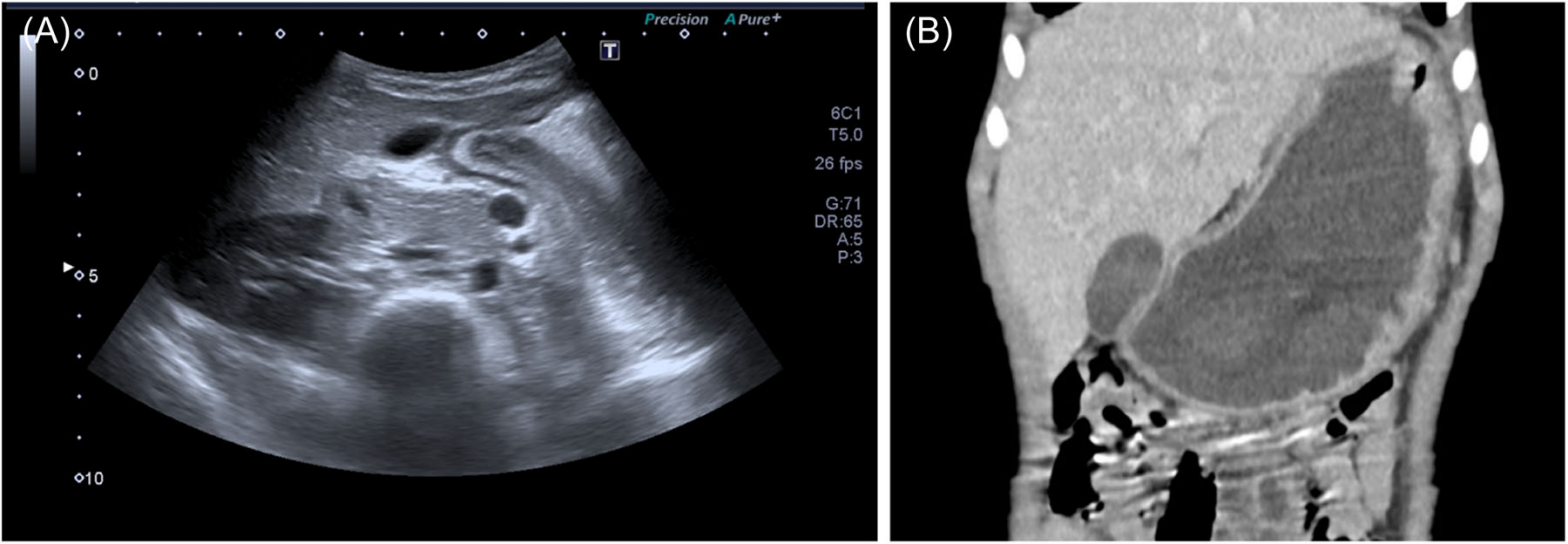
(A) Abdominal ultrasound with pyloric gastric wall thickening at diagnosis. (B) Abdominal CT scan showing gastric dilation, confirming the ultrasound diagnosis.

**Figure 2:**
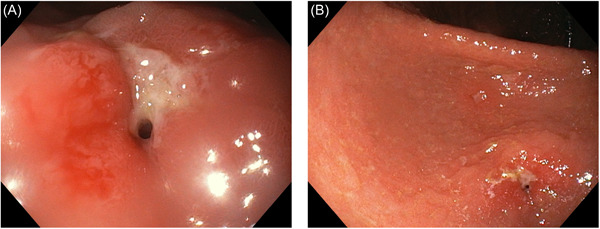
(A) Diagnostic upper endoscopy, (B) Third upper endoscopy, after corticosteroid treatment with persistent stenosis and ulcer of the pylorus.

**Figure 3:**
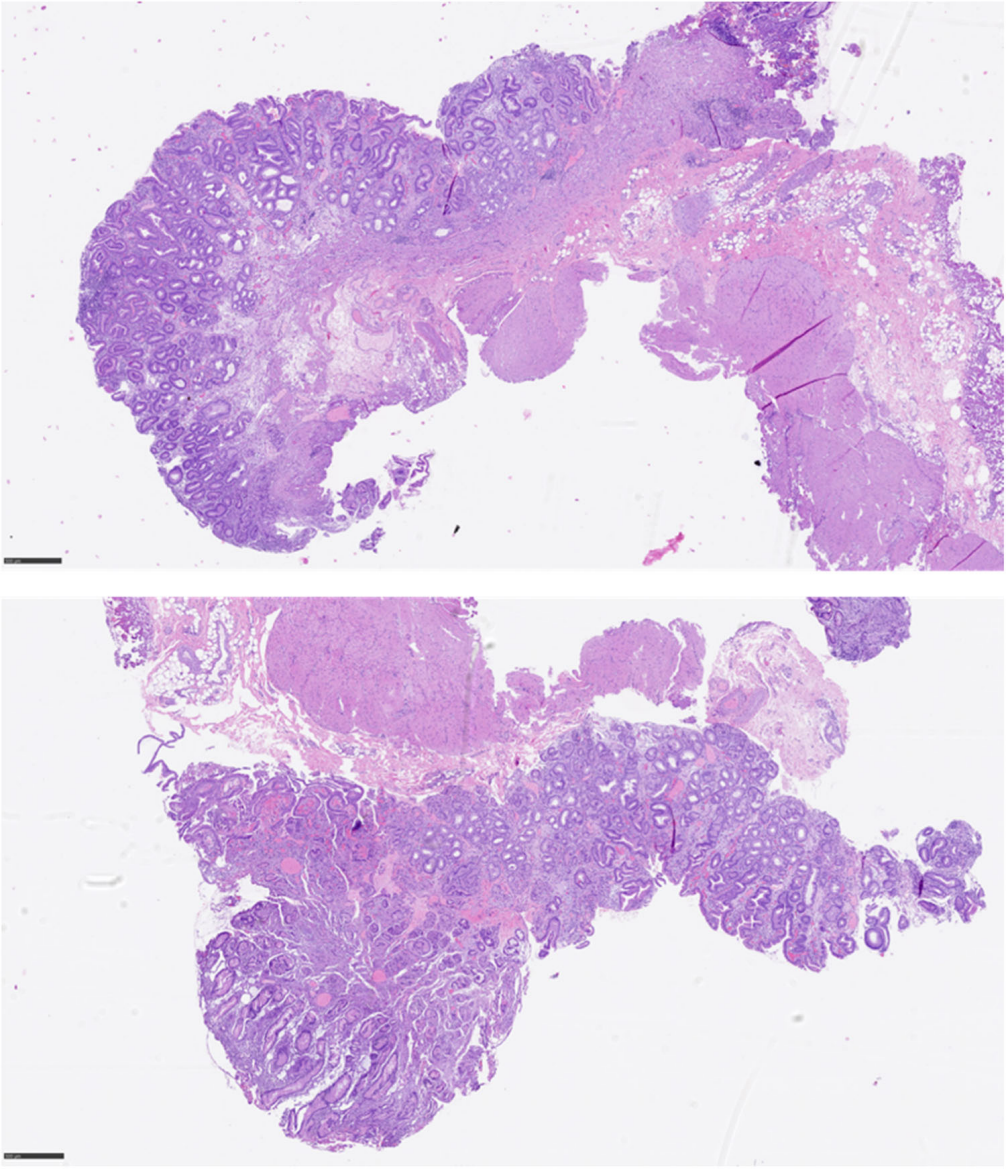
Gastric biopsy (H&E): Gastric mucosal wall with representation of the mucosal, submucosal and muscularis propria layer. Mucosa with reactive foveolar hyperplasia, congestion and edema. Foci of intestinal metaplasia are observed. Mucosal muscle with hyperplastic and disorganized features. Submucosa with edema and fibrous collagenosis. Muscular, with hyperplastic aspects. No fibrosis or elastosis is identified.

Afterwards, she underwent blood tests, including full blood count, liver, renal, and thyroid function tests, with normal results. Celiac disease was excluded, and fecal calprotectin levels were normal. Abdominal ultrassonography revealed isolated thickening of the pyloric gastric wall (Figures 1, 2, 3).

Upper endoscopy revealed abundant food content in the gastric fundus and severe stenosis of the pylorus that prevented progression to the duodenal bulb. Histopathological examination revealed marked foveolar hyperplasia and exocytosis of numerous lymphocytes. Tissue DNA analyses for Epstein‐Barr virus, cytomegalovirus, and Mycobacterium tuberculosis yielded negative results. An abdominal CT scan was performed, which excluded neoplastic lesions and confirmed the circumscribed involvement of the pyloric region.

She was hospitalized, and treatment with omeprazole and a polymeric diet was initiated because of complete solid food intolerance. Ten days later, a second upper endoscopy was performed with no improvement in the stenosis; subsequent treatment with oral betamethasone for 2 weeks did not contribute to clinical or endoscopic improvement.

Finaly, the patient underwent Heinke‐Miculicz pyloroplasty. Histopathological examination revealed mucosal reactive foveolar hyperplasia, submucosal collagenous fibrosis, and muscularis propria hyperplasia, which confirmed the diagnosis of late‐onset hypertrophic pyloric stenosis.

The postoperative period was uneventful, and the girl returned to solid oral food 3 days after surgery, with no complaints during the 6‐month follow‐up.

## 3 DISCUSSION

GOO is divided into two major groups according to etiology. Congenital causes such as antral diaphragm, pyloric atresia, and infantile hypertrophic pyloric stenosis usually become evident after birth or in the first 4–8 weeks of life. GOO can occur secondary to neoplasms (direct effect or compression), ingestion of caustic substances, Crohn's disease, gastric volvulus, and eosinophilic gastroenteritis. Besides these two causes, Jodhpur disease, which causes defects in pyloric motility without hypertrophy, can also lead to GOO.^2^, ^3^

The first case of late‐onset pyloric stenosis was reported in India in 1997 by *Sharma* et al., after which a few cases were reported worldwide. Patients may experience fullness after eating, epigastric pain, and vomiting, which can be particularly severe after meals. More severe cases may present with weight loss and dehydration, which sometimes leads to electrolyte disturbances.^2^

Most authors agree that late‐onset pyloric stenosis may correspond to the persistence of the infantile form that becomes clinically symptomatic only at a later stage when a triggering event exacerbates pyloric obstruction.^4^, ^5^

The diagnostic approach for a patient with uncoercive vomiting and abdominal distension should include blood analysis, electrolytes, and placement of a nasogastric tube to allow immediate gastric decompression. Abdominal radiography and ultrasound are the first‐line examinations used to identify gastric outlet obstruction, such as pyloric stenosis, and to exclude bowel obstruction. Abdominal CT or MRI is recommended to rule out extrinsic causes of GOO.^1^, ^5^

Owing to its rarity, data on treatment options are limited to case reports and case series.^4^ Some case reports have described the use of endoscopic balloon dilatation, botulinum toxin injection, or electrosurgical incisions without improvement.^1^, ^4^ Thus, pyloromyotomy is the gold standard treatment since it relieves gastric outlet obstruction and restores normal gastric emptying.^1^, ^6^

We chose surgical treatment because the patient had total solid food intolerance despite conservative treatment, and because of the lack of evidence supporting the use of botulinum toxin injection or electrosurgical incision.

## 4 CONCLUSION

Late‐onset pyloric stenosis is a rare condition that can cause significant morbidity, malnutrition, and weight loss. Early diagnosis and treatment are essential to restore oral feeding and weight gain. Our patient was effectively treated with pyloroplasty laparotomy, similar to other cases described in the literature.^1^ Further research is needed to understand the etiology of late‐onset hypertrophic pyloric stenosis and to identify potential risk factors for its development.

## CONFLICT OF INTEREST STATEMENT

The authors declare no conflicts of interest.
